# Deep learning-based quantification of T2-FLAIR mismatch sign: extending IDH mutation prediction in adult-type diffuse lower-grade glioma

**DOI:** 10.1007/s00330-025-11475-7

**Published:** 2025-03-07

**Authors:** Young Hun Jeon, Kyu Sung Choi, Kyung Hoon Lee, Seong Yun Jeong, Ji Ye Lee, Taehyuk Ham, Inpyeong Hwang, Roh-Eul Yoo, Koung Mi Kang, Tae Jin Yun, Seung Hong Choi, Ji-hoon Kim, Chul-Ho Sohn

**Affiliations:** 1 Department of Radiology, Seoul National University Hospital, Seoul, Korea; 2 Department of Radiology, Seoul National University College of Medicine, Seoul, Korea; 3 Department of Radiology, Kangbuk Samsung Hospital, Sungkyunkwan University School of Medicine, Seoul, Korea

**Keywords:** Glioma, Deep learning, Magnetic resonance imaging, Isocitrate dehydrogenase

## Abstract

**Objectives:**

To investigate the predictive value of the quantitative T2-FLAIR mismatch ratio (qT2FM) with fully automated tumor segmentation in adult-type diffuse lower-grade gliomas (LGGs).

**Materials and methods:**

This retrospective study included 218 consecutive patients (mean age, 47 years ± 15 [SD]; 125 males) diagnosed with adult-type diffuse LGG. The cohort was classified into IDH wild-type (IDHwt), IDH-mutant with 1p/19q-codeletion (IDHmut-Codel), and IDH-mutant without 1p/19q-codeletion (IDHmut-Noncodel) subtypes. Tumor masks were obtained using deep learning-based segmentation, and qT2FM was calculated from the differences in signal intensity ratios on T2 and FLAIR images. Multivariable logistic regression identified predictors for identifying IDHmut-Noncodel and IDH mutation status. Point-biserial correlations were analyzed between qualitative and quantitative T2FM, and median apparent diffusion coefficient (ADC) value. Diagnostic performance was evaluated with a receiver operating characteristic curve.

**Results:**

The IDHmut-Noncodel group had a higher qT2FM (0.37 ± 0.38, *p* = 0.004) than the IDHmut-Codel (0.24 ± 0.39) and IDHwt groups (0.07 ± 0.62). The qT2FM was the only independent imaging predictor for identifying IDHmut-Noncodel (OR = 3.43, 95% CI: 1.30–9.05, *p* = 0.01). Independent predictors of IDH mutation were younger age (*p* < 0.001), frontal lobe location (*p* = 0.007), cortical involvement (*p* < 0.001), and higher qT2FM (*p* = 0.034). The qT2FM significantly correlated with visual T2FM (vT2FM) and median ADC value. Adding qT2FM to vT2FM improved performance in identifying IDHmut-Noncodel (AUC 0.77, 95% CI: 0.70–0.82) and IDH mutation status (AUC 0.77, 95% CI: 0.71–0.83) than each parameter alone.

**Conclusion:**

The qT2FM ratio, derived from deep learning-based tumor segmentation, is a valuable predictor for identifying IDH mutation status and the IDHmut-Noncodel subtype in patients with adult-type diffuse LGG.

**Key Points:**

***Question*** *Does deep-learning-based quantification of the T2-FLAIR mismatch sign provide accurate prediction of IDH-mutant, 1p/19q non-codeleted astrocytomas and enhance identification of IDH mutation status*?

***Findings*** *Quantifying the T2-FLAIR mismatch sign with a fully automated segmentation tool achieved high accuracy in identifying IDH-mutant, 1p/19q non-codeleted astrocytomas, and enhanced IDH status prediction*.

***Clinical relevance*** *Integrating the qT2FM into clinical protocols enhances diagnostic precision and guides treatment strategies, underscoring the role of advanced imaging in neuro-oncology*.

## Introduction

World Health Organization (WHO) grades 2 and 3 gliomas, also known as lower-grade gliomas (LGGs), encompass a highly heterogeneous group of tumors that can be classified according to their different molecular and pathological features [1–3]. According to the WHO 2021 brain tumor classification, genetic information has become increasingly important for precisely diagnosing and developing individualized treatments for diffuse LGG patients [4]. The 2021 WHO classification includes three fundamental types of diffuse gliomas: (1) oligodendroglioma, isocitrate dehydrogenase (IDH)-mutant and 1p/19q-codeleted (IDHmut-Codel), (2) astrocytoma, IDH-mutant 1p/1q-noncodeleted (IDHmut-Noncodel), and (3) IDH wild-type glioma (IDHwt).

After Patel et al first reported the T2-fluid-attenuated inversion recovery (FLAIR) mismatch sign (T2FM) as a specific imaging biomarker for identifying adult patients with the IDHmut-Noncodel subtype, subsequent studies have consistently demonstrated the predictive value of this sign, recognizing its high specificity but low sensitivity [5–7]. Classically, the T2FM is defined as a complete or near-complete, homogeneous, hyperintense signal on T2-weighted imaging (T2WI) that combines a hypointense signal on FLAIR imaging with a hyperintense rim. In addition, recent studies have reported that FLAIR suppression within the tumor can be inhomogeneous, and the value of the hyperintense rim signal on FLAIR imaging, referred to as visual T2FM (vT2FM), has been emphasized to increase the sensitivity of the sign without compromising specificity [8, 9]. Malik et al attempted to classify adult-type diffuse gliomas and predict IDHmut-Noncodel status based on T2-FLAIR discordance volume grades [10]. However, inconsistencies in the definition of the vT2FM across these studies, along with low inter-reader reliability in distinguishing necrotic and cystic components from the tumor portion, pose significant challenges. Moreover, the reliance on visual assessment for measuring the T2FM volume limits its utility in clinical practice. These variability and methodological limitations underscore the need for standardized approaches to increase the clinical applicability of the T2FM sign.

Numerous studies have employed deep learning-based tumor segmentation programs to extract quantitative imaging biomarkers that predict glioma molecular subtypes [11–13]. Some recent studies have focused on quantifying the T2FM sign, utilizing methods that subtract tumor signals on T2 and FLAIR imaging to exploit residual signal signatures or by calculating the percentage volume of the T2FM [14, 15]. However, these methods require manual delineation of tumor masks and the use of specialized research software, which complicates their integration into clinical practice.

This study aims to quantify T2FM with fully automated tumor segmentation software and evaluate the predictive value of the quantitative T2FM (qT2FM) in identifying the IDHmut-Noncodel subtype and IDH mutation status in adult-type diffuse LGG patients.

## Materials and methods

### Study population

**Fig. 1:**
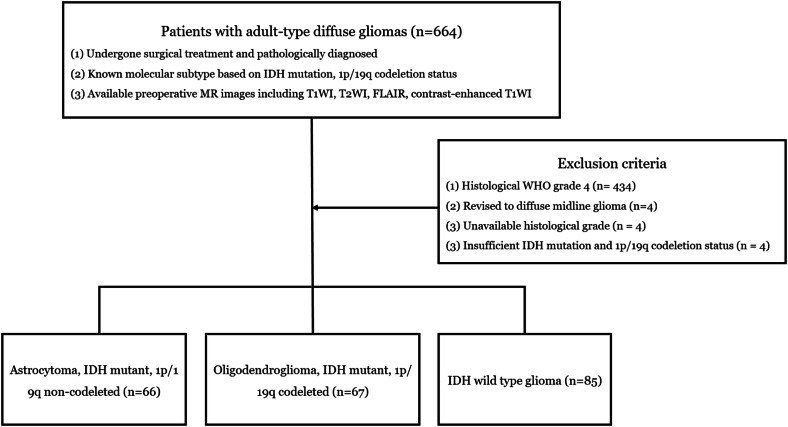
Flowchart of patient selection. IDH, isocitrate dehydrogenase; T1WI, T1-weighted image; FLAIR, fluid-attenuation inversion recovery

This retrospective study was approved by the institutional review board of X hospital, which waived the need for informed consent due to the retrospective nature of the study. Between January 2011 and August 2021, 664 adult patients newly diagnosed with adult-type diffuse glioma, according to the 2021 WHO brain tumor classification system, were recruited. The inclusion criteria were: (1) patients who underwent surgical treatment with a confirmed pathological diagnosis, (2) known molecular subtype based on IDH mutation and 1p/19q codeletion status, and (3) availability of preoperative MR images. The exclusion criteria were: (1) histological WHO grade 4 (*n* = 434), (2) revised diagnoses of diffuse midline glioma, H3 K27M-altered (*n* = 4), (3) unclassified histological WHO grades (*n* = 4), and (4) failure to identify molecular status, including IDH-mutant and 1p/19q-codeletion status (*n* = 4) (Fig. 1).

### Radiological data collection and image post-processing

All MR images were obtained on 3.0-T scanners with a 32-channel head coil and the participant in the supine position. The MRI acquisition protocols included the following sequences: axial T2WI, T1-weighted imaging (T1WI), FLAIR, and contrast-enhanced T1WI. Details are provided in Supplementary Table 1.

The freely available automated segmentation and volumetric measurement tool HD-GLIO (https://github.com/NeuroAI-HD/HD-GLIO) was used to produce tumor segmentation masks for the contrast-enhanced and non-contrast-enhanced T2/FLAIR signal abnormality portions on T1WI, T2WI, FLAIR, and contrast-enhanced T1WI [16, 17]. During this process, cystic or necrotic regions within the tumor were excluded. The FAST (FMRIB’s Automated Segmentation) tool within FSL was subsequently utilized to segment the normal-appearing white matter masks, which were used for normalization purposes [18]. All segmentations were visually inspected and manually corrected if needed (Supplementary Fig. 1). In addition, the regions of interest (ROIs) were then transferred to the co-registered DWI ma,p and the median value of apparent diffusion coefficient (ADC) was calculated.

### T2-FLAIR mismatch sign: qualitative and quantitative analysis

Three board-certified neuroradiologists (Y.H.J., K.H.L. and K.S.C., with 3 years, 4 years, and 7 years of neuroimaging experience) who were blinded to the patient’s clinical information, histopathology results, and molecular status, independently analyzed the preoperative MR images, and cases of discordance were resolved by consensus. Each reader evaluated the following image metrics: primary location of the tumor, multilobar involvement, cortical involvement, and the presence of vT2FM. For the primary location of the tumor, the neuroradiologists indicated whether the tumor was centered in the frontal lobe.

The T2FM was deemed positive when a tumor component showed a hyperintense signal on T2WI coupled with corresponding relative suppression on FLAIR imaging [5]. Then, according to the extent of the sign with respect to the tumor, the T2FM was classified as either classic T2FM, characterized by nearly complete concordance with the extent of the tumor; or partial T2FM, defined by correspondence to only a portion of the tumor. Visual T2FM was defined as a homogeneous, hyperintense T2 signal within a nonenhanced, solid tumor portion with corresponding FLAIR imaging suppression, not necessarily involving the entire tumor volume, and therefore encompasses both classic and partial T2FM [19]. Representative images of classic and partial T2FM are shown in Supplementary Fig. 2.

**Fig. 2:**
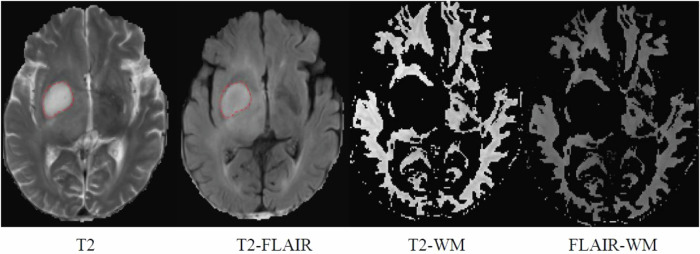
Quantification of the T2-FLAIR mismatch ratio. WM, white matter

For the quantitative analysis of T2FM, an ROI was delineated to encompass the entire hyperintense region within the T2/FLAIR binary mask, and the values were subsequently averaged. Another ROI was then delineated on the contralateral normal-appearing white matter (CNWM) mask on both T2-weighted and FLAIR images. The qT2FM value was calculated with the following equation [20]: qT2FM = [mean T2 signal intensity (SI) of the tumor/mean T2 SI of the CNWM] − [mean FLAIR SI of the tumor/mean FLAIR SI of the CNWM] (Fig. 2).

### Statistical analysis

Comparisons of categorical variables were performed with the chi-square test, and comparisons of quantitative variables were performed with Student’s *t*-test or one-way ANOVA. Uni- and multivariable logistic regression analyses were performed to identify predictors of IDH mutation status and the IDHmut-Noncodel subtype. Point biserial correlation was performed to identify the associations between vT2FM and qT2FM. Receiver operating characteristic (ROC) curve analysis was performed to calculate the area under the ROC curve (AUC) for the vT2FM and qT2FM. The AUCs of the individual and combined imaging parameters were compared with the DeLong method. Pearson correlation analysis was performed to evaluate the relationship between qT2FM and the median ADC. All the statistical analyses were performed with MedCalc software version 20.112 (MedCalc, Mariakerke, Belgium) and *p* < 0.05 (two-sided) was considered to indicate statistical significance.

## Results

### Characteristics of the study population

**Fig. 3:**
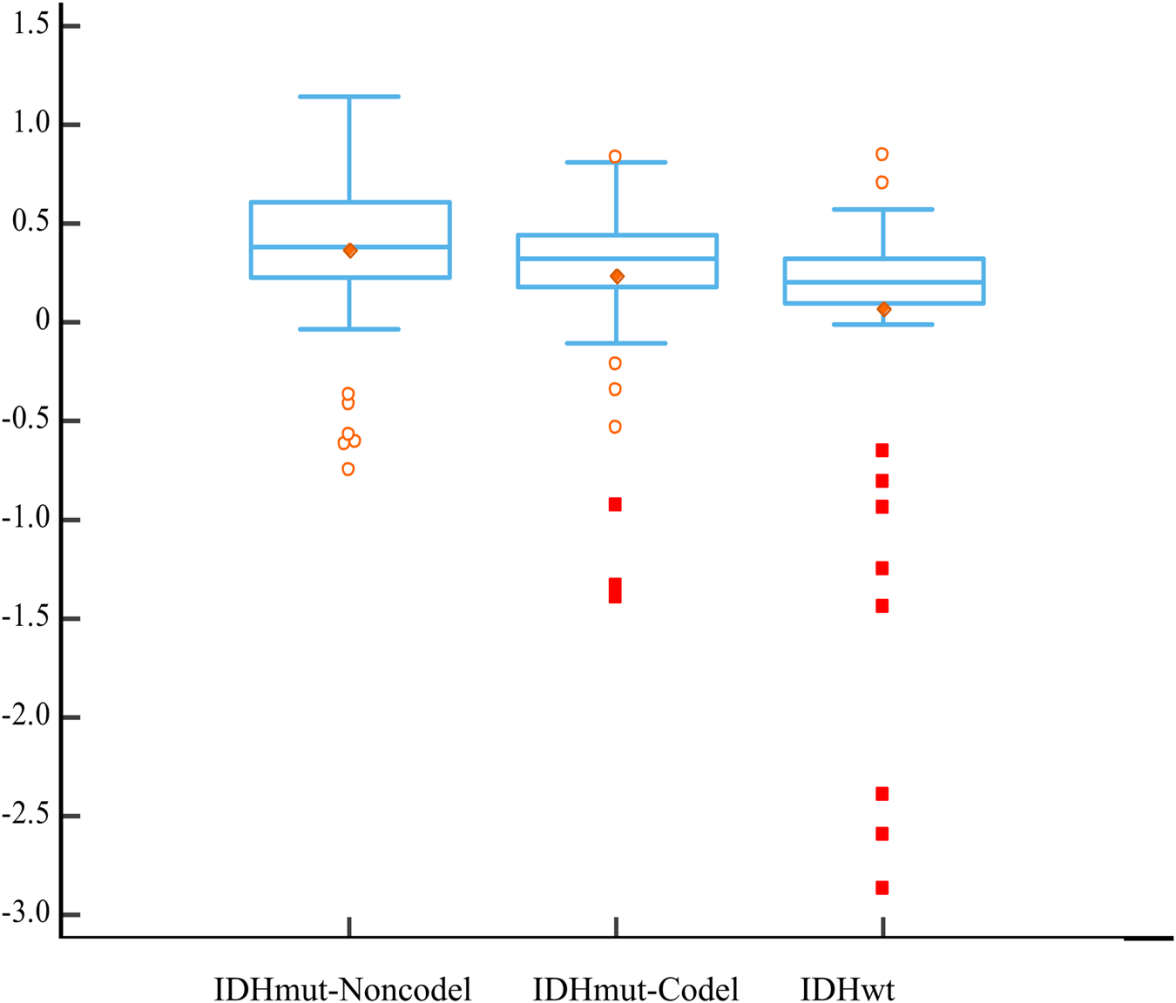
Associations between the qT2FM and molecular profiles. qT2FM, quantitative T2-FLAIR mismatch ratio; IDHmut-Noncodel, astrocytoma IDH-mutant and 1p/19q-noncodeleted; IDHmut-Codel, oligodendroglioma, IDH-mutant and 1p/19q-codeleted; IDHwt, IDH-wildtype glioma

A total of 218 patients (mean age, 47 years ± 15 [SD]; 125 male patients) were enrolled in the study. Among them, 66 were considered to have the IDHmut-Noncodel, 67 were considered to have the IDHmut-Codel, and 85 were considered to have the IDHwt (Table 1). Among the three adult-type diffuse LGG groups, after Bonferroni correction, significant differences were identified in age (*p* < 0.001), frontal lobe location (*p* < 0.001), cortical involvement (*p* < 0.001), vT2FM (*p* < 0.001), and qT2FM (*p* = 0.001). Classic T2FM was present in 20 patients with IDHmut-Noncodel and absent among IDHmut-Codel and IDHwt patients. In contrast, partial T2FM was identified across all types of diffuse LGG, with no significant difference between the IDHmut-Noncodel (37.9%, 25/66) and IDHmut-Codel (44.6%, 29/67) groups (*p* = 0.53). The IDHmut-Noncodel group had the highest qT2FM value (0.37 ± 0.38), followed by the IDHmut-Codel and IDHwt groups (0.24 ± 0.39 and 0.07 ± 0.62, respectively) (Fig. 3).

**Table 1:** Demographics of adult-type diffuse LGG patients

Characteristics	IDHmut-Noncodel (*n* = 66)	IDHmut-Codel (*n* = 67)	IDHwt (*n* = 85)	*p* value^†^
Age (y)^*^	39.8 ± 10.2	45.3 ± 12.7	54.7 ± 15.8	< 0.001
Female (%)	29 (43.9)	31 (47.1)	33 (38.8)	0.63
Frontal lobe location (%)	40 (60.6)	53 (79.1)	30 (35.3)	< 0.001
Multilobar involvement (%)	15 (22.7)	15 (22.4)	25 (29.4)	0.52
Cortical involvement (%)	56 (84.8)	65 (97.0)	46 (54.1)	< 0.001
vT2FM	45 (68.2)	29 (43.3)	11 (12.9)	< 0.001
Classic T2FM	20 (30.3)	0	0	< 0.001
Partial T2FM	25 (37.9)	29 (44.6)	11 (16.9)	< 0.001
qT2FM^*^	0.37 ± 0.38	0.24 ± 0.39	0.07 ± 0.62	0.001

Except where indicated, the data are the number of patients with percentages in parentheses

*IDHmut-Codel* IDH mutant 1p/19q codeleted oligodendroglioma, *IDHmut-Noncodel* IDH mutant 1p/19q noncodeleted, *IDHwt* IDH wild-type, *vT2FM* visual T2-FLAIR mismatch sign, *qT2FM* quantitative T2-FLAIR mismatch ratio

^*^ Data are mean and standard deviations

^†^ Comparison among groups

### Differentiation of IDHmut-Noncodel from other diffuse LGGs

In univariable analysis, age (*p* < 0.001) and qT2FM (*p* = 0.003) were significant differentiators of the IDHmut-Noncodel subtype from other diffuse LGGs. Both age (odds ratio [OR] = 0.95; 95% CI: 0.92–0.97, *p* < 0.001) and qT2FM (OR = 3.43; 95% CI: 1.30–9.05, *p* = 0.013) remained significant predictors in the multivariable analysis (Table 2).

**Table 2:** Multivariable logistic regression for predicting IDHmut-Noncodel subtype

	Univariable		Multivariable	
Variable	Odds ratio (95% CI)	*p*	Odds ratio (95% CI)	*p*
Age	0.94 (0.92, 0.97)	< 0.001	0.95 (0.92, 0.97)	< 0.001
Female	1.07 (0.60, 1.93)	0.80		
Frontal lobe location	1.28 (0.71, 2.30)	0.41		
Cortical involvement	2.07 (0.97, 4.43)	0.06		
Multilobar involvement	0.82 (0.42, 1.62)	0.58		
qT2FM	4.91 (1.71, 14.13)	0.006	3.43 (1.30, 9.05)	0.013
Median ADC	1.00 (0.999, 1.001)	0.46		

Data in parentheses are 95% confidence intervals

*qT2FM* quantitative T2-FLAIR mismatch ratio, *ADC* apparent diffusion coefficient

### Prediction of IDH mutation status

In univariable analysis, age (*p* < 0.001), frontal lobe location (*p* < 0.001), cortical involvement (*p* < 0.001), and qT2FM (*p* = 0.004) were significant predictors of IDH mutation status among adult-type diffuse LGG patients. In the multivariable analysis, age (OR = 0.93; 95% CI: 0.90–0.95, *p* < 0.001), frontal lobe location (OR = 2.65; 95% CI: 1.30–5.38, *p* = 0.007), cortical involvement (OR = 11.00; 95% CI: 4.35–27.79, *p* < 0.001), and qT2FM (OR = 2.75; 95% CI: 1.08–7.00, *p* = 0.034) remained significant predictors of the IDH mutation status (Table 3).

**Table 3:** Multivariable logistic regression for IDH mutation status

	Univariable		Multivariable	
Variable	Odds ratio (95% CI)	*p*	Odds ratio (95% CI)	*p*
Age	0.94 (0.92, 0.96)	< 0.001	0.93 (0.90, 0.95)	< 0.001
Female	1.27 (0.81, 2.38)	0.39		
Frontal lobe location	4.22 (2.36, 7.53)	< 0.001	2.65 (1.30, 5.38)	0.007
Cortical involvement	8.48 (4.08, 17.61)	< 0.001	11.00 (4.35, 27.79)	< 0.001
Multilobar involvement	0.70 (0.38, 1.30)	0.26		
qT2FM	2.81 (1.40, 5.62)	0.004	2.75 (1.08, 7.00)	0.034
Median ADC	0.99 (0.998, 1.006)	0.50		

Data in parentheses are 95% confidence intervals

*qT2FM* quantitative T2-FLAIR mismatch ratio, *ADC* apparent diffusion coefficient

### Comparing diagnostic performance: visual vs quantitative T2-FLAR mismatch ratio

The qT2FM was significantly correlated with the presence of the vT2FM (point biserial correlation [rpb] = 0.21, *p* = 0.002) and partial T2FM (*r* = 0.20, *p* = 0.003). However, there was no significant correlation between the qT2FM and the presence of the classic T2FM (*r* = 0.05, *p* = 0.45) (Supplementary Table 2).

**Fig. 4:**
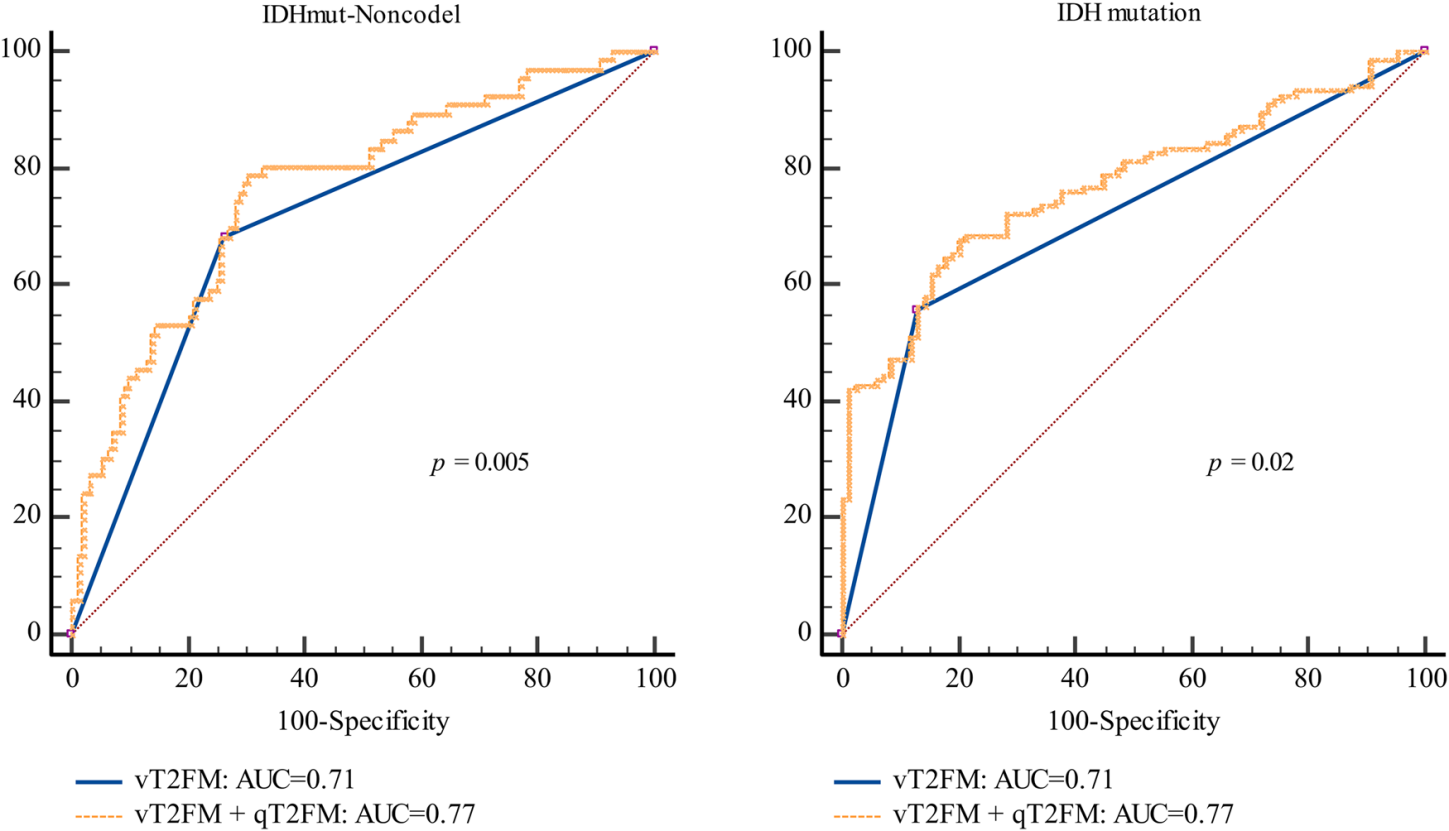
Receiver operating characteristic (ROC) curves for predicting IDHmut-Noncodel and IDH mutation status. IDH, isocitrate dehydrogenase; IDHmut-Noncodel, astrocytoma, IDH-mutant and 1p/19q-noncodeleted; vT2FM, visual T2-FLAIR mismatch sign; qT2FM, quantitative T2-FLAIR mismatch ratio

In distinguishing IDHmut-Noncodel from the other two adult-type diffuse LGGs, the AUC of the vT2FM was 0.71 (95% CI: 0.64–0.77), with a sensitivity, specificity, PPV, and NPV of 52.9%, 84.2%, 68.2%, and 73.7%, respectively, while the AUC of the qT2FM was 0.68 (95% CI: 0.61–0.74). The combination of the qT2FM and vT2FM was significantly better (AUC, 0.77; 95% CI: 0.70–0.82; *p* = 0.005) than the single parameters alone in identifying the IDHmut-Noncodel subtype. There was no significant difference between the AUCs of the single parameters (*p* = 0.50) (Fig. 4).

In predicting IDH mutation among adult-type diffuse LGG patients, the AUCs of the qT2FM and vT2FM were 0.69 (95% CI: 0.62–0.75) and 0.71 (95% CI: 0.65–0.77), respectively, but the difference between the two was not significant. In contrast, the AUCs of the combination of qT2FM and vT2FM were significantly different from those of the single parameters alone (AUC, 0.77; 95% CI: 0.71–0.83; *p* = 0.004).

### Correlation between the qT2FM and the ADC

**Fig. 5:**
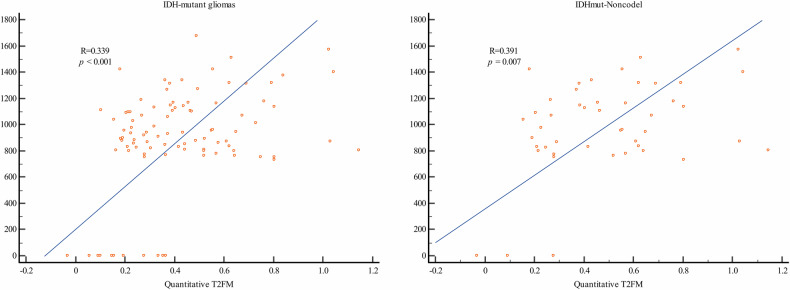
Scatter diagram of qT2FM ratio and median ADC for patients with IDH-mutant LGG. Analysis of all subtypes of IDH-mutant gliomas and a separate analysis for the subset of patients with the IDHmut-Noncodel subtype were performed. qT2FM, quantitative T2-FLAIR mismatch ratio; ADC, apparent diffusion coefficient; LGG, lower-grade glioma; IDH, isocitrate dehydrogenase; IDHmut-Noncodel, astrocytoma, IDH-mutant and 1p/19q-noncodeleted; IDHmut-Codel, oligodendroglioma, IDH-mutant and 1p/19q-codeleted; IDHwt, IDH-wildtype glioma

A significant correlation was found between qT2FM and median ADC for patients with IDH-mutant glioma (*r* = 0.339, *p* < 0.001) (Fig. 5). To exclude the confounding effect of the IDHmut-Codel subtype, which frequently demonstrates a lower ADC compared to the IDHmut-Noncodel subtype, a subgroup analysis was performed exclusively on patients with the IDHmut-Noncodel subtype. Similar results were obtained, with a significant correlation between the qT2FM and the median ADC (*r* = 0.391, *p* = 0.007).

## Discussion

This study is the first to use deep learning-based quantification of the T2-FLAIR mismatch sign (qT2FM) with a fully automated tumor segmentation tool to identify the IDHmut-Noncodel subtype among adult-type diffuse LGG patients. Our approach provides more accurate and reproducible results than manual methods by capturing the entire tumor volume and addressing tumor heterogeneity. The qT2FM was shown to be an independent imaging predictor for both IDHmut-Noncodel and IDH mutation status in multivariable analysis, further validating its predictive capability. Moreover, when combined with the visual T2-FLAIR mismatch ratio (vT2FM), the qT2FM tended to better predict IDH mutation status than the vT2FM alone. Additionally, the positive correlation between qT2FM and median ADC highlights its pathophysiological relevance and supports its diagnostic utility in neuro-oncology.

While several studies have validated the clinical utility of the T2FM for identifying the IDHmut-Noncodel subtype with nearly 100% positive predictive value and specificity, this sign has demonstrated poor sensitivity, with only a small fraction of IDHmut-Noncodel tumors exhibiting the T2FM [5, 7, 21]. Recent studies have focused on enhancing the diagnostic sensitivity for the IDHmut-Noncodel subtype by modifying and extending the T2FM criteria. The updated definition now accounts for cases where the T2FM is visible only in parts of the tumor or where there is a hyperintense rim on FLAIR imaging, regardless of the signals from the tumor component on T2WI [8–10]. In this study, the expanded definition of the T2FM was applied, allowing the quantification of the relative signal intensities on both T2WI and FLAIR imaging across the entire tumor, and the results demonstrated that the T2FM was a unique imaging predictor for the IDHmut-Noncodel subtype (OR = 3.43, 95% CI: 1.30–9.05, *p* = 0.013). Previous studies have attempted to utilize the proportion of tumor volume demonstrating FLAIR suppression as the T2FM in classifying LGG patients. Malik et al [10] classified tumors with a T2-FLAIR mismatch volume of more than 50% as the IDHmut-Noncodel subtype, those with less than 25% mismatch volumes as the IDHmut-Codel subtype, and those with matched T2-FLAIR volumes as the IDHwt subtype. This study’s findings were on par with those of the previous study, demonstrating that the qT2FM value is likely on a spectrum in adult-type diffuse LGG, allowing for patient subcategorization based on molecular subtype [10]. Notably, adult-type diffuse LGG patients with the IDH mutation had significantly greater qT2FM values than those with the IDHwt subtype (IDHmut-Noncodel vs IDHmut-Codel vs IDHwt; 0.37 ± 0.38 vs 0.24 ± 0.39 vs 0.07 ± 0.62, *p* = 0.001). Furthermore, similar to previous studies, some patients with the IDHmut-Codel subtype also presented with the partial T2FM sign [10, 19, 22]. However, there is persistent controversy regarding the qualitative evaluation of the T2FM sign because the appearance of a positive mismatch sign significantly varies depending on the inversion time of T2 FLAIR imaging [23].

In this study, the qT2FM sign was confirmed as a predictive marker for the IDH mutation, extending beyond its utility in identifying the IDHmut-Noncodel subtype. Lee et al [19] reported that the partial T2FM sign was a specific imaging predictor for IDH mutation status in WHO grade 4 glioma patients. Consistent with these findings, our study demonstrated that when combined with cortical involvement and frontal lobe location, the qT2FM is a significant imaging predictor. Furthermore, the qT2FM reduces the potential for false-positive results that may arise from the presence of a partial T2FM sign caused by cystic or necrotic changes.

To the best of our knowledge, this study is the first to utilize a fully automated tumor segmentation program to develop a simple quantitative imaging predictor for the IDHmut-Noncodel subtype. Our findings differ from those of Lee et al [20], who calculated the T2FM ratio using manually drawn ROIs and concluded that it was not a significant indicator for the IDHmut-Noncodel subtype. Manual quantification of the T2FM may not accurately reflect the visual T2FM due to tumor heterogeneity, as it is limited to a portion of the tumor. In a previous study on volumetric analysis of T2FM [14], Cho et al used a semi-automated thresholding method for tumor segmentation and digital subtraction maps between T2-weighted and FLAIR images to calculate the T2-FLAIR volume percentage. They found that ≥ 42% T2-FLAIR mismatch volume was specifically indicative of the IDHmut-Noncodel subtype, highlighting the importance of tumor subregion volumes showing mismatch rather than the degree of FLAIR suppression. In contrast, our quantitative approach, utilizing a deep learning-based fully automated tumor segmentation, captures the entire tumor signal on both T2-weighted and FLAIR imaging. This approach could potentially be integrated into real clinical practice and significantly enhance diagnostic accuracy in identifying IDHmut-Noncodel and IDH mutation status, offering a more comprehensive evaluation than manual or semi-automated methods.

Although few patients presented with the classic T2FM, previous studies have indicated that compared with the IDHwt subtype, the IDHmut-Noncodel subtype is characterized by histopathologically lower cellular density and lower ADC values [6, 24–26]. Consistent with these findings, our results also revealed significantly higher qT2FM values in IDH-mutant gliomas than in IDHwt gliomas. Furthermore, correlation analysis revealed a positive correlation between the vT2FM and qT2FM (*R* = 0.214, *p* = 0.002), suggesting that even in the absence of the T2FM, the IDHmut-Noncodel subtype might exhibit distinctive tumor microenvironment features, such as microcystic changes and enlarged intracellular spaces.

Although the histopathological features of the IDHmut-Noncodel subtype have not been fully elucidated, recent studies have reported that IDH-mutant LGG patients presenting with the T2FM often exhibit microcystic changes and enlarged intracellular spaces. These changes can influence the diffusion of water molecules, which may contribute to the manifestation of the T2FM [25–27]. The T2FM can be identified visually in patients for whom these tumor microenvironment features are prominent. Furthermore, employing automated tumor segmentation for quantitative analysis can capture certain pathological changes associated with the T2FM, thus improving its sensitivity in predicting IDH mutation status.

Region-based ADC measurements have been effectively used to identify IDH mutation status in adult-type diffuse LGG patients and have excellent interobserver agreement and great diagnostic accuracy [1, 6, 28]. Furthermore, Foltyn et al demonstrated a significant positive correlation between the T2FM and the median ADC in IDH-mutant LGG [6]. Intriguingly, recent studies have indicated that patients with the IDHmut-Noncodel subtype without the T2FM also exhibit a significantly greater ADC than either IDHwt or IDHmut-Codel patients [24]. Our findings align with those of prior studies, revealing a significant positive correlation between the qT2FM and the median ADC in IDH-mutant LGG, including IDHmut-Noncodel LGG. This finding suggests that, in addition to the ADC, qT2FM serves not only as a radiological marker for the IDHmut-Noncodel subtype but also closely reflects the histopathological pathophysiology of the tumor, encompassing the tumor microenvironment. DL-based radiomics can be used to identify a superset of ring-like signs such as T2FM, both quantitative and qualitative.

There are several limitations to this study. First, the study employs a single-center retrospective design and is therefore vulnerable to selection bias. Thus, a large cohort, multicenter study is needed to improve the generalizability of our results. Second, quantification requires additional calculations via image processing, which hampers the clinical application of the qT2FM in real-world settings.

In conclusion, quantification of the T2-FLAIR mismatch ratio, derived from deep learning-based tumor segmentation, provides valuable predictive information for identifying IDH mutation status and identifying the IDHmut-Noncodel subtype in patients with adult-type diffuse LGG. Integrating the qT2FM into clinical protocols may increase diagnostic accuracy and influence treatment strategies, emphasizing the increasing relevance of advanced imaging techniques in neuro-oncology.

## Supplementary information

ELECTRONIC SUPPLEMENTARY MATERIAL

## Abbreviations

ADC: Apparent diffusion coefficient
IDH: Isocitrate dehydrogenase
IDHmut-Codel: Oligodendroglioma, IDH-mutant and 1p/19q-codeleted
IDHmut-Noncodel: Astrocytoma, IDH-mutant and 1p/19q-noncodeleted
IDHwt: IDH-wildtype glioma
LGG: Lower-grade glioma
qT2FM: Quantitative T2-FLAIR mismatch ratio
ROI: Region of interest
vT2FM: Visual T2-FLAIR mismatch sign
WHO: World Health Organization

## References

[1] Maynard J, Okuchi S, Wastling S et al (2020) World Health Organization Grade II/III glioma molecular status: prediction by MRI morphologic features and apparent diffusion coefficient. Radiology 296:111–121 32315266 10.1148/radiol.2020191832

[2] Jo J, van den Bent MJ, Nabors B, Wen PY, Schiff D (2022) Surveillance imaging frequency in adult patients with lower-grade (WHO grade 2 and 3) gliomas. Neuro Oncol 24:1035–1047 35137214 10.1093/neuonc/noac031 PMC9248400

[3] Brat DJ, Verhaak RG, Aldape KD et al (2015) Comprehensive, integrative genomic analysis of diffuse lower-grade gliomas. N Engl J Med 372:2481–2498 26061751 10.1056/NEJMoa1402121 PMC4530011

[4] Louis DN, Perry A, Wesseling P et al (2021) The 2021 WHO classification of tumors of the central nervous system: a summary. Neuro Oncol 23:1231–1251 34185076 10.1093/neuonc/noab106 PMC8328013

[5] Patel SH, Poisson LM, Brat DJ et al (2017) T2-FLAIR mismatch, an imaging biomarker for IDH and 1p/19q status in lower-grade gliomas: a TCGA/TCIA project. Clin Cancer Res 23:6078–6085 28751449 10.1158/1078-0432.CCR-17-0560

[6] Foltyn M, Nieto Taborda KN, Neuberger U et al (2020) T2/FLAIR-mismatch sign for noninvasive detection of IDH-mutant 1p/19q non-codeleted gliomas: validity and pathophysiology. Neurooncol Adv 2:vdaa004 32642675 10.1093/noajnl/vdaa004 PMC7212872

[7] Park SI, Suh CH, Guenette JP, Huang RY, Kim HS (2021) The T2-FLAIR mismatch sign as a predictor of IDH-mutant, 1p/19q-noncodeleted lower-grade gliomas: a systematic review and diagnostic meta-analysis. Eur Radiol 31:5289–5299 33409784 10.1007/s00330-020-07467-4

[8] Throckmorton P, Graber JJ (2020) T2-FLAIR mismatch in isocitrate dehydrogenase mutant astrocytomas: variability and evolution. Neurology 95:e1582–e1589 32690782 10.1212/WNL.0000000000010324

[9] Li M, Ren X, Chen X et al (2022) Combining hyperintense FLAIR rim and radiological features in identifying IDH mutant 1p/19q non-codeleted lower-grade glioma. Eur Radiol 32:3869–3879 35079884 10.1007/s00330-021-08500-w

[10] Malik P, Soliman R, Chen YA et al (2024) Patterns of T2-FLAIR discordance across a cohort of adult-type diffuse gliomas and deviations from the classic T2-FLAIR mismatch sign. Neuroradiology 66:521–530 38347151 10.1007/s00234-024-03297-z

[11] van der Voort SR, Incekara F, Wijnenga MMJ et al (2023) Combined molecular subtyping, grading, and segmentation of glioma using multi-task deep learning. Neuro Oncol 25:279–289 35788352 10.1093/neuonc/noac166 PMC9925710

[12] Jian A, Jang K, Manuguerra M, Liu S, Magnussen J, Di Ieva A (2021) Machine learning for the prediction of molecular markers in glioma on magnetic resonance imaging: a systematic review and meta-analysis. Neurosurgery 89:31–44 33826716 10.1093/neuros/nyab103

[13] Kikuchi K, Togao O, Yamashita K et al (2024) Comparison of diagnostic performance of radiologist- and AI-based assessments of T2-FLAIR mismatch sign and quantitative assessment using synthetic MRI in the differential diagnosis between astrocytoma, IDH-mutant and oligodendroglioma, IDH-mutant and 1p/19q-codeleted. Neuroradiology 66:333–341 38224343 10.1007/s00234-024-03288-0 PMC10859342

[14] Cho NS, Sanvito F, Le VL et al (2024) Quantification of T2-FLAIR mismatch in nonenhancing diffuse gliomas using digital subtraction. AJNR Am J Neuroradiol 45:188–197 38238098 10.3174/ajnr.A8094 PMC11285991

[15] Mohammed S, Ravikumar V, Warner E et al (2022) Quantifying T2-FLAIR mismatch using geographically weighted regression and predicting molecular status in lower-grade gliomas. AJNR Am J Neuroradiol 43:33–39 34764084 10.3174/ajnr.A7341 PMC8757555

[16] Isensee F, Schell M, Pflueger I et al (2019) Automated brain extraction of multisequence MRI using artificial neural networks. Human Brain Mapp 40:4952–4964 10.1002/hbm.24750 PMC6865732 31403237

[17] Kickingereder P, Isensee F, Tursunova I et al (2019) Automated quantitative tumour response assessment of MRI in neuro-oncology with artificial neural networks: a multicentre, retrospective study. Lancet Oncol 20:728–740 30952559 10.1016/S1470-2045(19)30098-1

[18] Zhang Y, Brady M, Smith S (2001) Segmentation of brain MR images through a hidden Markov random field model and the expectation-maximization algorithm. IEEE Trans Med Imaging 20:45–57 11293691 10.1109/42.906424

[19] Lee MD, Patel SH, Mohan S et al (2023) Association of partial T2-FLAIR mismatch sign and isocitrate dehydrogenase mutation in WHO grade 4 gliomas: results from the ReSPOND consortium. Neuroradiology 65:1343–1352 37468750 10.1007/s00234-023-03196-9 PMC11058040

[20] Lee MK, Park JE, Jo Y, Park SY, Kim SJ, Kim HS (2020) Advanced imaging parameters improve the prediction of diffuse lower-grade gliomas subtype, IDH mutant with no 1p19q codeletion: added value to the T2/FLAIR mismatch sign. Eur Radiol 30:844–854 31446467 10.1007/s00330-019-06395-2

[21] Jain R, Johnson DR, Patel SH et al (2020) “Real world” use of a highly reliable imaging sign: “T2-FLAIR mismatch” for identification of IDH mutant astrocytomas. Neuro Oncol 22:936–943 32064507 10.1093/neuonc/noaa041 PMC7339896

[22] Patel SH, Batchala PP, Muttikkal TJE et al (2021) Fluid attenuation in non-contrast-enhancing tumor (nCET): an MRI Marker for Isocitrate Dehydrogenase (IDH) mutation in Glioblastoma. J Neurooncol 152:523–531 33661425 10.1007/s11060-021-03720-y

[23] Kinoshita M, Arita H, Takahashi M et al (2020) Impact of inversion time for FLAIR acquisition on the T2-FLAIR mismatch detectability for IDH-mutant, non-CODEL astrocytomas. Front Oncol 10:596448 33520709 10.3389/fonc.2020.596448 PMC7841010

[24] Aliotta E, Dutta SW, Feng X et al (2020) Automated apparent diffusion coefficient analysis for genotype prediction in lower grade glioma: association with the T2-FLAIR mismatch sign. J Neurooncol 149:325–335 32909115 10.1007/s11060-020-03611-8

[25] Deguchi S, Oishi T, Mitsuya K et al (2020) Clinicopathological analysis of T2-FLAIR mismatch sign in lower-grade gliomas. Sci Rep 10:10113 32572107 10.1038/s41598-020-67244-7 PMC7308392

[26] Yamashita S, Takeshima H, Kadota Y et al (2022) T2-fluid-attenuated inversion recovery mismatch sign in lower grade gliomas: correlation with pathological and molecular findings. Brain Tumor Pathol 39:88–98 35482260 10.1007/s10014-022-00433-6

[27] Fujita Y, Nagashima H, Tanaka K et al (2021) The histopathologic and radiologic features of T2-FLAIR mismatch sign in IDH-mutant 1p/19q non-codeleted astrocytomas. World Neurosurg 149:e253–e260 33610870 10.1016/j.wneu.2021.02.042

[28] Do YA, Cho SJ, Choi BS et al (2022) Predictive accuracy of T2-FLAIR mismatch sign for the IDH-mutant, 1p/19q noncodeleted low-grade glioma: an updated systematic review and meta-analysis. Neurooncol Adv 4:vdac010 35198981 10.1093/noajnl/vdac010 PMC8859831

