# Supplementary material for: Deep learning-based quantification of T2-FLAIR mismatch sign: extending IDH mutation prediction in adult-type diffuse lower-grade glioma
Source: Eur Radiol. 2025 Mar 7;35(9):5193–202. doi: 10.1007/s00330-025-11475-7 (PMC12350433; doi:10.1007/s00330-025-11475-7)
Supplement: Supplementary file 1 — ELECTRONIC SUPPLEMENTARY MATERIAL [file 330_2025_11475_MOESM1_ESM.pdf]

Deep Learning-Based Quantification of T2-FLAIR Mismatch Sign: Extending IDH Mutation Prediction in Adult-Type Diffuse Lower-Grade Glioma

Electronic Supplemental Material

Eur Radiol (2025) Jeon YH, Choi KS, Lee KH, et al.

Supplementary Table 1. MRI scan parameters

		Siemens (Magnetom Skyra 3T)	Philips (Ingenia CX 3T)	GE (Discovery 750w 3T)
**Axial T2WI**	TR/TE (ms)	3100/100	3000/110	6400/100
	Flip angle (degrees)	150	90	160
	Number of averages	2	1	1
	Matrix size	512x512	512x350	448x448
	Slice thickness (mm)	5	5	5
	Interslice gap (mm)	1	1	1
	FOV (mm)	220x220	220x220	220x220
	Acceleration	GRAPPA 2	SENSE 1.5	-
	Echo train length	23	18	18
	Acquisition time (sec)	142	168	202
**Axial T2 FLAIR**	TR/TE (ms)	8000/105	9000/110	9000/131
	Flip angle (degrees)	150	90	160
	Number of averages	1	1	1
	Matrix size	384x269	340x192	320x256
	Slice thickness (mm)	5	5 (concatenation 4)	5
	Interslice gap (mm)	1	1	1
	Field of view (mm)	220x220	220x220	220x220
	Acceleration	GRAPPA 2	SENSE 2	ARC 2
	Echo train length	23	32	21
	Acquisition time (sec)	145	180	153
**Axial T1WI**	TR/TE (ms)	1800/9	1800/10	1750/22
	Flip angle (degrees)	150	90	111
	Number of averages	1	1	1
	Matrix size	320x240	320x240	352x256
	Slice thickness (mm)	5	5	5
	Interslice gap (mm)	1	1	1
	Field of view (mm)	220x220	220x220	220x220
	Acceleration	GRAPPA 2	SENSE 2.5	-
	Echo train length	9	4	8
	Acquisition time (sec)	135	146	109

Abbreviations: T1WI=T1-weighted imaging, T2WI=T2-weighted imaging, FLAIR=fluid-attenuated inversion recovery, TR=repetition time, TE=echo time, FA=flip angle, NEX=number of excitations, FOV=field of view

Supplementary Table 2. Correlation between qualitative and quantitative T2-FLAIR mismatch sign

	Pearson correlation coefficient (point biserial)	*P* value
qT2FM vs. pT2FM	0.199 (-0.062, 0.196)	0.003
qT2FM vs. vT2FM	0.214 (0.083, 0.337)	0.002

Note.–Data in parentheses are 95% confidence intervals.

Abbreviations: qT2FM= quantitative T2-FLAIR mismatch ratio, pT2FM= partial T2-FLAIR mismatch sign, vT2FM= visual T2-FLAIR mismatch sign

Supplementary Figure 1. Manual Correction of Segmentation Failures in HD-GLIO: Two Case Examples

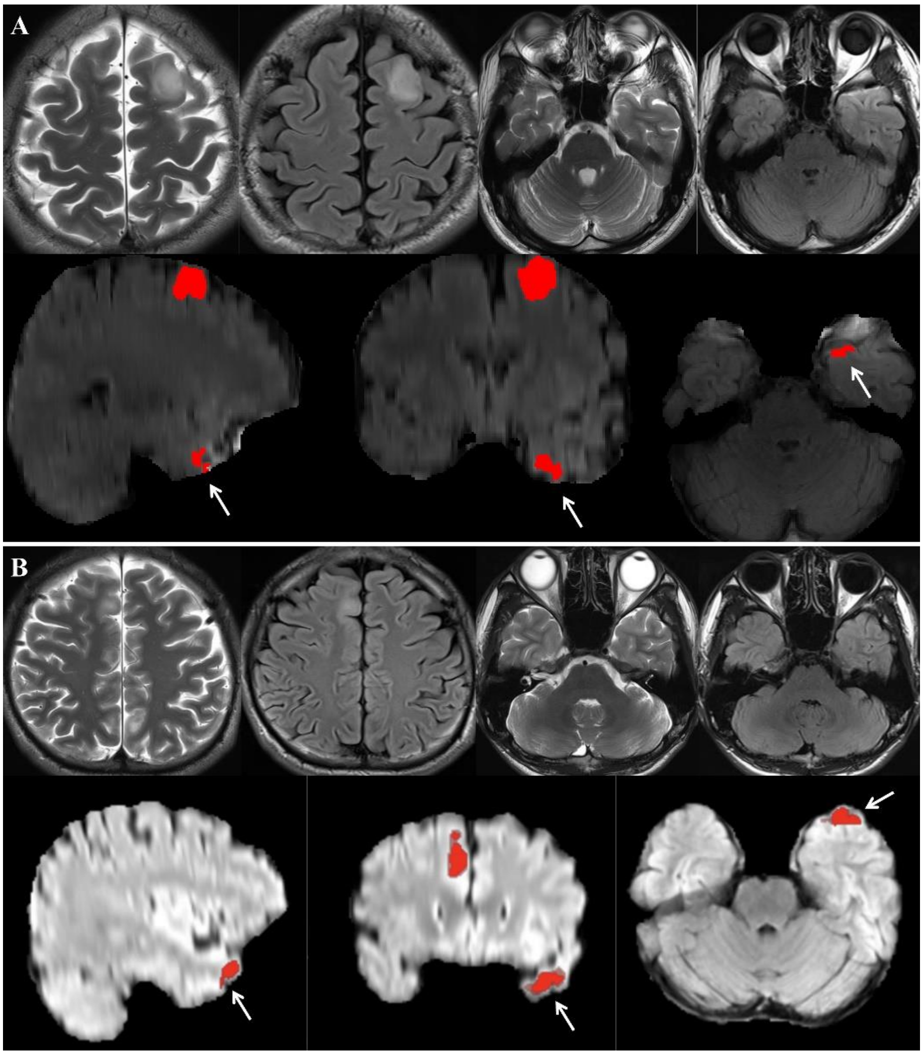

(A) A 1.8 cm T2 hyperintense mass involving the left frontal lobe was confirmed as oligodendroglioma, IDH-mutant, 1p/19q-codeleted. However, during automated tumor segmentation using HD-GLIO, the program delineated an additional false-positive tumor mask (arrow) at the left temporal pole. T2-weighted imaging (T2WI) revealed severe susceptibility artifacts with architectural distortion and hyperintensity at the left temporal convexity, resulting in this segmentation failure.

(B) A 2.0 cm ill-defined T2 hyperintense mass involving the right frontal lobe was confirmed as astrocytoma, IDH-mutant, 1p/19q-intact. HD-GLIO generated a false-positive tumor mask (arrow) at the left temporal pole. T2WI demonstrated a subtle hyperintense lesion at the left temporal pole, likely caused by partial volume averaging artifacts, leading to segmentation failure.

Abbreviations: IDH=Isocitrate dehydrogenase, FLAIR= fluid-attenuated inversion recovery

Supplementary Figure 2. Representative images of the classic and partial T2-FLAIR mismatch signs

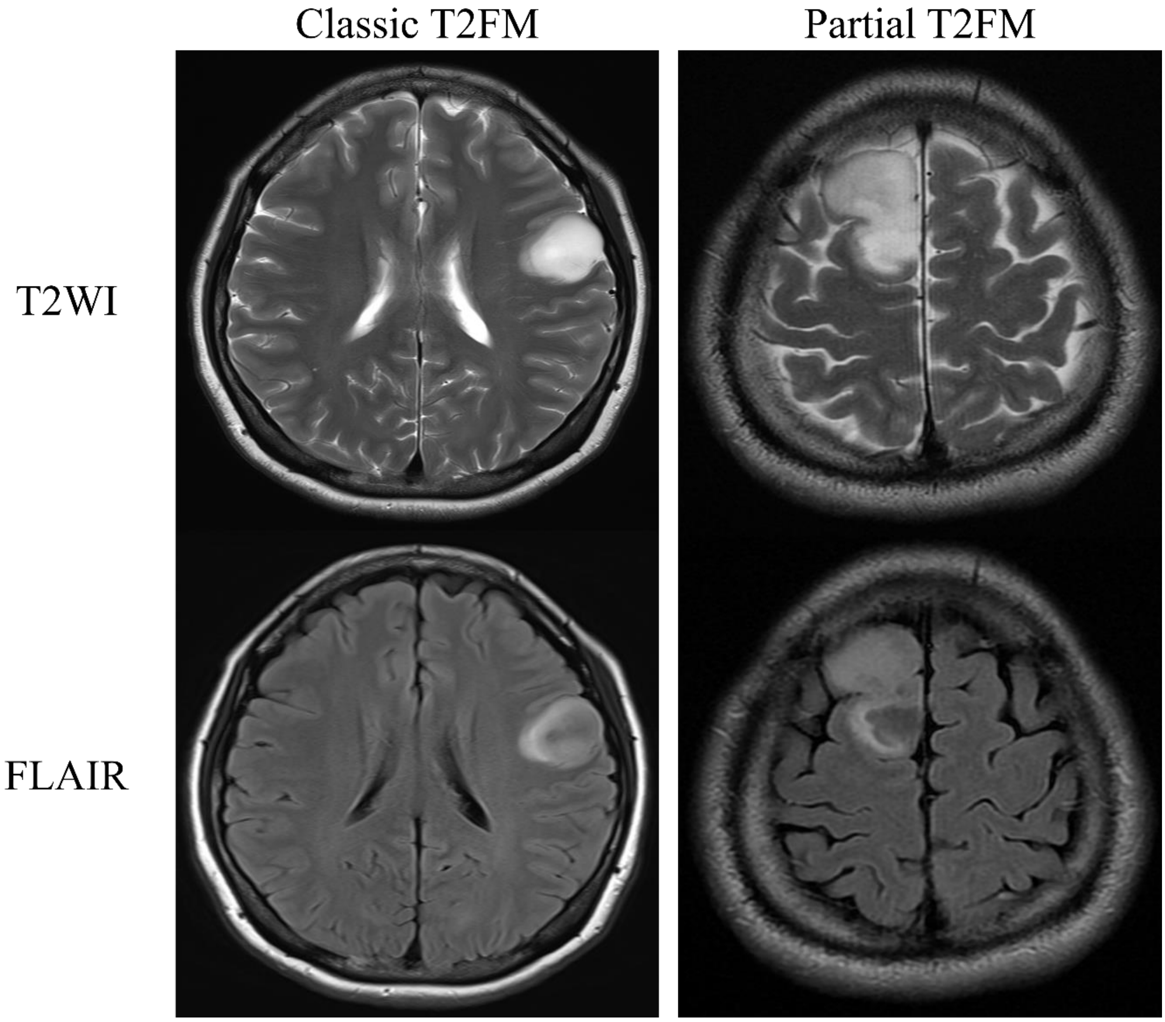

Abbreviations: T2FM= T2-FLAIR mismatch, T2WI= T2-weighted imaging, FLAIR= fluid-attenuated inversion recovery

#
